# John Purdue Gray

**Published:** 1887-01

**Authors:** 


### *JOHN PURDUE GRAY.*

"One of the founders and an ex-president of the New York State Medical Association, died after a long illness at Utica, N. Y., November 29, 1886, aged 61 years. He was the Medical Superintendent of the State Lunatic Asylum at Utica, having assumed the duties of that position in September, 1850, and it is no exaggeration to say that he made this institution the most favorably known in the world. As an alienist he was accepted as an authority, and almost invariably appeared as an expert in all cases of importance. His testimony is credited with being lucid, analytical and eminently impartial. When Guiteau was tried for the assassination of President Garfield he was chief of the experts summoned to Washington by the prosecution. His testimony followed that of all the other experts, in order that a careful review of the case might go to the jury at the close with all the weight of his ability and reputation. He did not see 'the slightest shadow of insanity' in Guiteau, and with this conclusion the nation fully coincided.

"On March 16, 1882, an unsuccessful attempt was made to assassinate Dr. Gray in Utica. He was in his office reading his mail, having just returned from Washington, when Henry Reimshaw entered and fired a shot at him from a revolver. The ball struck the doctor's left cheek bone, passed through the right cheek and lodged in the wall. The wound was not a serious one and the doctor soon recovered. Reimshaw was employed in Bagg's Hotel, and for eighteen months had been under the delusion that he was an ambassador sent from Heaven to shoot Dr Gray. He had never been a patient in the asylum, but had taught Dr. Gray's children to swim.

"As a man, Dr. Gray was an enemy of sham and pretence, possessed of a judicial mind, and master of himself in the most trying situations; was thorough in the investigation of all medico-legal points bearing upon the particular case in hand, and always commanded the respect of his judges for the honesty of his opinions. He was active in the profession, defending its best interests; broad and liberal in his views, very approachable, and an especial friend to the meritorious, struggling practitioner. In the relations of life he was beyond censure, and died regretted in the community where he was best known."

To him is due the credit for most of the advances made in the last few years for the care of the insane. By medical teaching and example he taught the world that the insane were sick persons and not wild beasts, that careful medical treatment and good nursing were as essential to this class of sick as any other. The improvement in the asylum life of this class of patients, their amusement and occupation, were all largely due to his instruction. Many times has he had come back to him from others, as new and advanced ideas, methods which he had long before recommended and introduced. At the time of his death he was professor of psychology in Bellevue Hospital Medical College and in the Albany Medical College, and the editor of the *American Journal of Insanity.* He was also president of the psychological section of the International Medical Congress.

